# Precision enzyme discovery through targeted mining of metagenomic data

**DOI:** 10.1007/s13659-023-00426-8

**Published:** 2024-01-11

**Authors:** Shohreh Ariaeenejad, Javad Gharechahi, Mehdi Foroozandeh Shahraki, Fereshteh Fallah Atanaki, Jian-Lin Han, Xue-Zhi Ding, Falk Hildebrand, Mohammad Bahram, Kaveh Kavousi, Ghasem Hosseini Salekdeh

**Affiliations:** 1 Department of Systems and Synthetic Biology, Agricultural Biotechnology Research Institute of Iran (ABRII), Agricultural Research Education and Extension Organization (AREEO), Karaj, Iran; 2 Human Genetics Research Center, Baqiyatallah University of Medical Sciences, Tehran, Iran; 3 Laboratory of Complex Biological Systems and Bioinformatics (CBB), Institute of Biochemistry and Biophysics (IBB), University of Tehran, Tehran, Iran; 4 Livestock Genetics Program, International Livestock Research, Institute (ILRI), Nairobi, 00100 Kenya; 5 CAAS-ILRI Joint Laboratory On Livestock and Forage Genetic Resources, Institute of Animal Science, Chinese Academy of Agricultural Sciences (CAAS), Beijing, 100193 China; 6 Key Laboratory of Yak Breeding Engineering, Lanzhou Institute of Husbandry and Pharmaceutical Sciences, Chinese Academy of Agricultural Sciences (CAAS), Lanzhou, 730050 China; 7 Gut Microbes and Health, Quadram Institute Bioscience, Norwich, Norfolk UK; 8 Digital Biology, Earlham Institute, Norwich, Norfolk UK; 9 Department of Ecology, Swedish University of Agricultural Sciences, Ulls Väg 16, 756 51 Uppsala, Sweden; 10 Department of Botany, Institute of Ecology and Earth Sciences, University of Tartu, 40 Lai St, Tartu, Estonia; 11 Faculty of Natural Sciences, Macquarie University, Sydney, NSW Australia

**Keywords:** Metagenomics, Enzyme bioprospecting, Functional-based screening, Sequence-based screening, Protein structure prediction, Natural products

## Abstract

Metagenomics has opened new avenues for exploring the genetic potential of uncultured microorganisms, which may serve as promising sources of enzymes and natural products for industrial applications. Identifying enzymes with improved catalytic properties from the vast amount of available metagenomic data poses a significant challenge that demands the development of novel computational and functional screening tools. The catalytic properties of all enzymes are primarily dictated by their structures, which are predominantly determined by their amino acid sequences. However, this aspect has not been fully considered in the enzyme bioprospecting processes. With the accumulating number of available enzyme sequences and the increasing demand for discovering novel biocatalysts, structural and functional modeling can be employed to identify potential enzymes with novel catalytic properties. Recent efforts to discover new polysaccharide-degrading enzymes from rumen metagenome data using homology-based searches and machine learning-based models have shown significant promise. Here, we will explore various computational approaches that can be employed to screen and shortlist metagenome-derived enzymes as potential biocatalyst candidates, in conjunction with the wet lab analytical methods traditionally used for enzyme characterization.

## Introduction

Enzymes are becoming increasingly valuable for the development of industrial catalysts due to their ability to significantly enhance the rate of biochemical reactions. High efficiency and selectivity are crucial characteristics for choosing enzymes for commercial applications in bio-catalysis, biofuels, and bioremediation. It is not surprising that microbial enzymes make up most commercial enzymes (88%) used in industry, as they offer several advantages over plant- or animal-derived enzymes [1, 2]. These advantages include higher stability, greater production yield, easier optimization, and increased cost-effectiveness in the industrial applications [3, 4]. Despite their advantages, the number of commercially available microbial enzymes is limited. The use of traditional culture-dependent microbiological methods to screen natural diversity for unknown enzymes is a common approach to obtaining appropriate microbial biocatalysts with desirable properties. This approach involves enriching microorganisms from environmental samples in the presence of appropriate substrates, isolating pure cultures, and screening microbial isolates to ultimately identify enzymes of interest [5]. While this method has proven successful in identifying many commercially available enzymes, more than 99% of microorganisms present in environmental samples cannot be cultured using standard laboratory techniques [6]. The consequence of this limitation is a potential loss of microbial diversity and the opportunity to discover novel enzymes with desired catalytic properties.

Advances in next-generation sequencing technologies have made it possible to access the genome sequences of all microorganisms present in an environment, without the need for their isolation and cultivation. The process of subjecting the DNA extracted from a community of microorganisms recovered from an environmental sample to whole-genome shotgun sequencing is referred to as metagenomics [7, 8]. The method enables direct sequencing of environmental DNA (eDNA) to explore community diversity, functional activities, and interactions of microorganisms inhabiting a specific environment [9]. Metagenomic sequences can be assembled de novo into contigs that represent the genomic segments of microorganisms from which they originate. This allows us to access the coding sequences of enzymes from uncultured microorganisms and predict their functional potentials under specific environmental conditions.

This approach can be used to explore the genomic sequences of unknown microorganisms residing in an environment for the discovery of novel enzymes with improved catalytic properties [10, 11]. It is demonstrated by the steadily increased number of predicted protein-coding sequences from metagenome sequencing of microbial communities obtained from diverse environments. Despite the current annotation pipelines, a significant portion of these sequences remains functionally uncharacterized, leaving many of them as unknown entities. The challenge is further magnified during the identification of a particular enzyme with an enhanced catalytic property. To tackle this problem, there is a growing interest in developing novel computational tools that can model the catalytic properties of enzymes by utilizing shared structural and functional features preserved in their amino acid sequences.

The current experimental approaches for identifying and characterizing new enzymes are limited in terms of speed and throughput, resulting in a gap between the numbers of discovered sequences and enzymes that are experimentally characterized with respect to their catalytic properties [12]. Assaying the activity of these enzyme sequences, especially the large number of novel enzymes predicted from metagenomic sequences, is impractical. Additionally, to realize the industrial application of an enzyme, it needs to be designed to meet specific process requirements [13]. All these limitations highlight the importance of physicochemical and structural features to be considered when searching for enzymes with properties suitable for a specific industrial or biotechnological application. The current approaches for *in-silico* enzyme discovery rely on the properties of enzymes inferred from phylogenetic analyses, sequence similarity searches, genomic positional information, three-dimensional (3D) structural modeling, and predictions based on machine learning [14]. Phylogenetic analyses can help infer the common ancestral origin of enzymes with shared catalytic properties. The sequence divergence that occurs during natural evolution can introduce variability in catalytic properties. The inclusion of sequences from catalytically efficient enzymes in phylogenetic analyses can help to identify distantly related sequences that may possess novel functional activities. Deep learning models can be employed to predict the structures of target enzymes by utilizing multiple sequence alignments and protein contact maps of many metagenomic sequences [15, 16]. Sequence similarity networks are valuable tools for identifying new candidate protein subfamily clusters by leveraging pairwise sequence similarities [17]. Genomic context provides important information regarding substrates, cofactors, bioactivity, and other co-regulated genes associated with the target enzymes [14]. For example, enzymes targeting a specific glycan substrate can be identified based on their genomic localization in polysaccharide utilization loci [18, 19].

Most deciphered 3D structures to date pertain to the enzymes isolated from cultured organisms, leaving limited experimental evidence regarding the structural characteristics of enzymes discovered through metagenome sequencing. Predicting protein function from structural data presents a significant challenge due to numerous instances of highly conserved protein folds that catalyze different reactions [14]. However, performing protein structural similarity searches is a crucial step in narrowing down the sequences that encode enzymes of interest from metagenome datasets. This approach aids in gaining functional insights into unknown sequences, eliminating the need for costly wet lab experiments. When combined with sequence homology-based searches, this approach possesses significant power in shortlisting specific enzymes within vast metagenomic datasets. While the 3D structure of enzymes plays a crucial role in their functionality, its utilization in the *in-silico* bioprospecting of novel enzymes remains limited.

Although several review papers have investigated the application of function-based, homology-based, and machine-learning-based methods to identify, predict, and annotate enzyme-encoding sequences in metagenome data [14, 20–24], there is a lack of knowledge regarding the integration of structural data into annotation pipelines. In this review, we provide an overview of various approaches and pipelines currently employed to explore metagenomic sequences for the discovery of new enzymes. In particular, we will emphasize the significance of incorporating structural data when searching for potential biocatalysts and natural bioproducts in large metagenome datasets.

## Bioprospecting of novel enzymes from environmental samples

Microbes residing in diverse environments, including soil, hydrothermal vents, saline or alkaline lakes, acid mine drainage, permafrost, hot springs, wastewater treatment sludges, and animal guts, offer the potential for discovering novel enzymatic processes [9]. The microbial communities inhabiting these environments are typically complex in terms of their composition and abundance. It is also worth noting that most members within these communities may not possess desired functions. As a result, comprehensive screening approaches are necessary to identify the desired enzymatic process in a complex environmental sample. Traditional screening approaches involved cultivating microorganisms under defined culture conditions and subsequently screening for microbial clones that exhibit the function of interest. As noted earlier, this approach is unable to capture all microbial diversity in the environment, a phenomenon known as the “great plate count anomaly” [25].

**Fig. 1:**
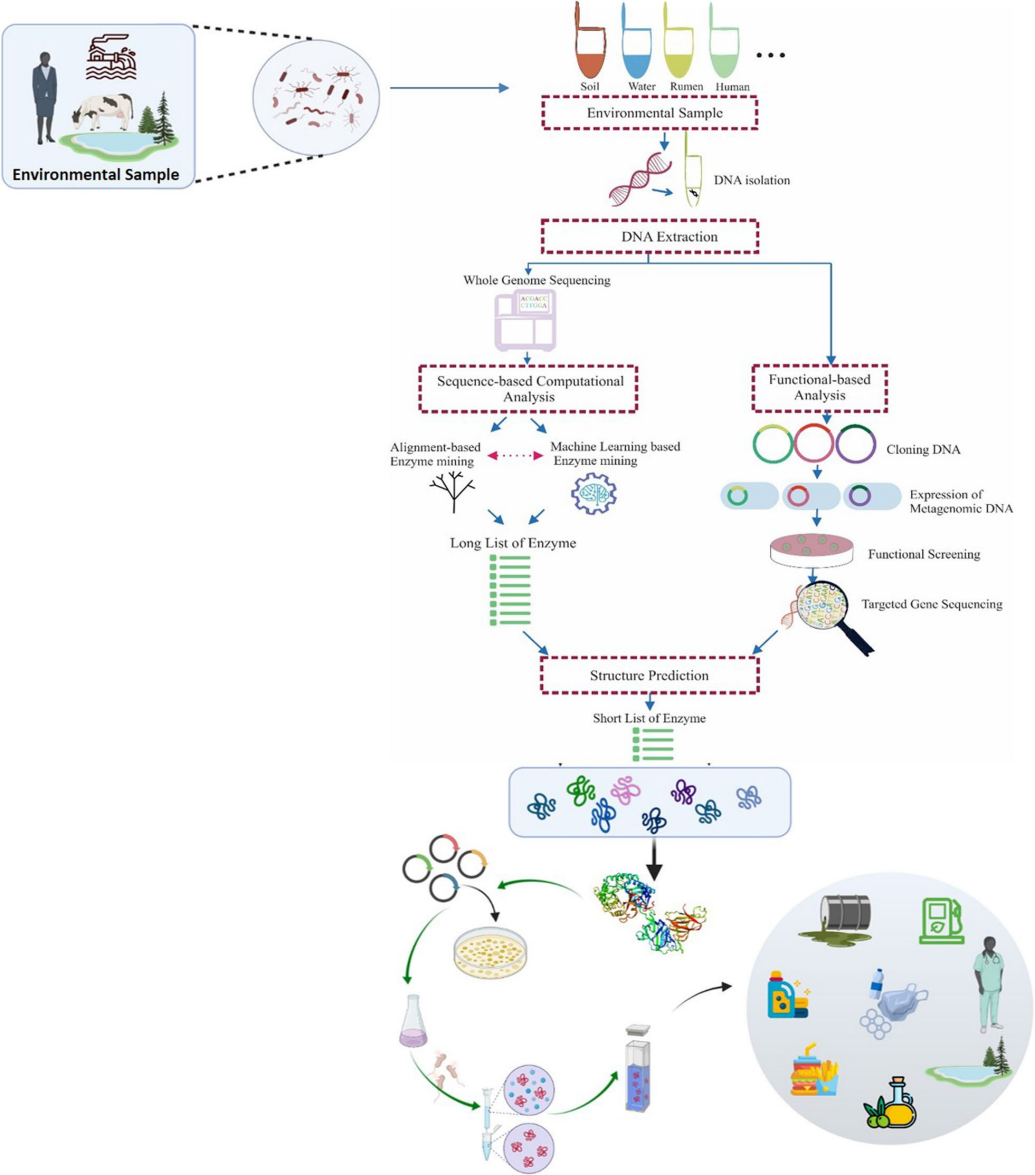
Culture-independent screening methods for mining novel enzymes from environmental samples. Both function-based and sequence-based methods can benefit from the information gained through structural analysis to refine the initial list of candidate enzymes

To address the limitations associated with culture-based screening approaches, culture-independent methods were introduced. These methods are classified into two major approaches: functional-based screening (FBS) and sequence-based screening (SBS). Figure 1 provides a comprehensive summary of culture-independent screening approaches commonly used to search for novel biocatalysts in eDNA.

### Function-based screening (FBS)

FBS involves direct cloning of eDNA libraries into suitable vectors, followed by functional screening in surrogate hosts such as *E. coli* [26]. During the screening process, the clones are examined to identify enzymes capable of utilizing a specific substrate or producing a specific product. After identifying the clones with desired functional properties, the DNA encoding for the function of interest is sequenced to identify the gene responsible for observed enzymatic activity. It is important to note that while this method has the potential to screen thousands of eDNA libraries, there are several limitations. The method becomes labor-intensive due to the necessity of analyzing a large number of clones to encompass the entire range of microorganisms present in an environmental sample [27]. The lack of expression of DNA originating from distantly related microorganisms in the surrogate host may result in a reduced representation of significant diversity in the screened samples. In addition, if the desired function relies on the coordinated activity of multiple enzymes, it is essential for all the encoding genes clustered in the same genomic region to be recovered in a single clone [28].

One significant advantage of FBS is its independence from prior knowledge of gene sequences or even the existence of such enzymes. Nevertheless, screening a typical metagenome library necessitates the evaluation of a substantial number of clones. As the complexity of the library increases, this process becomes labor-intensive, time-consuming, and expensive. To expedite the screening process, robotic instruments have been developed, allowing for efficient processing of complex eDNA libraries at a rate of up to 10 million per day. This method enables assaying for a single substrate, thereby reducing the chance of identifying highly promiscuous and multi-functional enzymes [20]. The success of identifying a target enzyme depends on several factors, including the assay method, gene size, gene abundance in the metagenomic sample, host-vector system, and the efficiency of gene expression in the surrogate host [26, 29].

FBS has been widely used for screening novel enzymes, including cellulase [30], esterase [31, 32], carboxylesterase [33], and lipases [34] from diverse environmental sources. In a recent study using activity-based screening through complementary sequence and structure analyses, a novel esterase was isolated by investigating lipolytic enzymes from a compost metagenome library [35]. The same approach was used to identify four thermo-alkaliphilic glycosyl hydrolases from wheat straw-degrading microbial consortia [36]. These enzymes hold the potential for utilization in lignocellulosic biomass-degrading cocktails.

### Sequence-based screening (SBS)

The conventional methods for enzyme discovery are generally laborious, costly, resource-intensive, and time-consuming, with no guarantee of success. Due to the availability of a vast number of manually curated protein sequences as well as experimentally characterized enzymes in public databases, the development of novel computational approaches has become imperative to leverage this information in the enzyme discovery process [2, 24]. Specifically, these valuable resources can be utilized to construct machine-learning models that can aid in biocatalyst prospecting. SBS methods expedite the discovery of novel enzymes while minimizing resource usage and achieving a higher success rate.

Prior to the emergence of metagenome sequencing, SBS relied predominantly on the design of primers or probes derived from conserved regions of known enzymes to amplify or screen eDNA libraries in the quest for novel enzyme sequences. This method allows for the identification of novel candidate variants of known enzyme sequences but does not possess the capability to discover entirely new enzymes [27]. Metagenome sequencing has revolutionized the field by enabling the sequencing of complete DNA extracted from a specific environmental sample [37]. Considering this capability, we can now delve into the genetic constituent of every microorganism present in any environment and gain access to all coding sequences, enabling the exploration of any enzymes. The primary challenge associated with this approach lies in accurately annotating the coding sequences predicted in metagenomic sequences. Presently, the annotation process relies on sequence homology searches against known genes or pathways available in public databases. However, the process lacks optimal efficiency, with over 40% of protein-coding sequences remaining unannotated and labeled as unknown or hypothetical. The situation becomes more complex when searching for an enzyme that catalyzes a specific hydrolytic or biosynthetic reaction within a vast number of protein-coding sequences predicted in a metagenomic dataset. The search for a new enzyme through bioprospecting of metagenomic sequences can be carried out by using two general approaches: de novo and reference-based, depending on the availability of known enzyme families [14]. The de novo discovery of new biocatalysts using SBS is challenging, particularly when there is no prior knowledge about the function of interest. Recent studies suggest that predicting protein structures and comparing structural models using residue-residue contact maps can be used to model unknown structures and assist in identifying new biocatalysts in metagenomic datasets [38, 39]. Reference-based methods can be employed when there is existing knowledge about the members of a specific class of enzymes, but the search is for enzymes with distinct functionality. Identifying new enzymes may be less challenging when there are experimentally characterized members, compared to situations where there is a lack of prior knowledge about enzyme function and structure. Robinson et al. [14] proposed a roadmap for metagenomic enzyme discovery, termed “enzyme expansion”, which aims to discover enzymes with novel catalysts, substrate specificities, and reaction conditions. Considering that both the reference-based and “enzyme expansion” methods aim to identify enzymes with novel catalytic properties, we have integrated them as a reference-based approach. It is clear that de novo and reference-based methods of enzyme discovery can effectively leverage homology-based (HB), structural-based (SB), and machine learning-based (MLB) analyses.

#### Homology-based (HB) analysis

Sequence homology search can be used to identify sequences that are closely or distantly related to known enzymes. The method holds significant potential for discovering novel functional homologs of known enzymes [2]. The approach is not only effective in expanding homologs of known enzymes but also capable of searching for enzymes with unique functions. The search is typically conducted on the sequences deposited in public databases, including Pfam, RefSeq, UniPort, and NCBI non-redundant protein (NCBI-nr). Due to inadequate annotations in most publicly available databases, the search results may include hits that are incorrectly annotated [15], thus the results must be manually curated. Several tools have been developed to facilitate the search for closely related sequences, including BLAST [40], DIAMOND [41], and USEARCH [42]. Search algorithms based on either profile Hidden Markov Models (HMMs) such as HMMER [43] or position-specific scoring matrices such as PSI-BLAST [44] can be utilized to identify distantly related sequences. In addition, automated annotation platforms such as MetaHMM [45] and ANASTASIA [46] have been developed to facilitate enzyme discovery through homology-based search. The success of the method hinges on selecting the appropriate target database for the homology search and ensuring the accuracy of annotations for the sequences within that database.

The sequence homology search can be utilized to narrow down a large set of ORFs predicted in a complex metagenome dataset, specifically focusing on enzymes with unique functional characteristics, including thermostability, pH stability, specific activity, and more [47]. In a study by Elbehery et al. [48], HB analysis was carried out to identify two antibiotic resistance genes from the metagenome of Atlantis II Deep Red Sea brine pool. Protein-coding sequences were annotated against sequences deposited in the Comprehensive Antibiotic Resistance Database (CARD, https://card.mcmaster.ca/) using BLASTx, leading to the successful identification of two ORFs encoding a class A beta-lactamase and an aminoglycoside-3’ phosphotransferase. The properties of these enzymes were further elucidated through 3D structure prediction. Garg et al. [49] applied HB analysis to identify a novel cellulase (Cel5R) from a soil metagenome. The enzyme was subsequently characterized for its salt- and heat-stable properties. The 3D structure of the enzyme was determined through crystallography. In the landmark study leading to the development of the ANASTASIA platform, a novel esterase named EstDZ4 was mined in a hot spring metagenome [46]. The HB analysis proved successful in identifying EstDZ4, which showed thermostable properties, making it a promising candidate for biotechnological application. The result of this study demonstrated the efficacy of *in-silico* analyses in identifying enzymes that exhibit remote similarity to known sequences.

#### Machine learning-based (MLB) analysis

In HB analysis, it is assumed that homologous sequences share similar functions. However, it is important to acknowledge that there can be exceptions to this rule, where two closely related sequences may possess different functions. Consequently, relying solely on sequence homology may lead to wrongly interpreted or overlooked functional variations. To address these limitations, additional analyses and experimental validations are often necessary to accurately determine functional attributes of closely related sequences. To incorporate additional features in function prediction, methods that leverage MLB analysis can be employed. MLB analysis utilizes advanced algorithms and models to learn patterns and relationships from various data sources, including sequence information, structural properties, physicochemical characteristics, and functional annotations [50, 51]. By considering a broader range of features, MLB analysis can enhance the accuracy and specificity of function prediction, enabling the identification of enzymes with unique and diverse functional characteristics. Moreover, MLB algorithms can detect non-linear relationships and patterns in the data, increasing the likelihood of discovering novel enzymes compared to HB analysis. MLB analysis has demonstrated its effectiveness in uncovering hidden functional relationships and facilitating the discovery of novel biocatalysts with specific catalytic activities and desirable properties.

Several MLB approaches have been developed for the functional classification of the enzymes. Table 1 lists some of the methods that utilize MLB models for the annotation of protein sequences and the prediction of EC numbers. It is important to note that while the methods presented in Table 1 primarily focus on identifying mono-functional enzymes, there are specialized tools such as mlDEEPre [52] that enable the prediction of both multi-functional and mono-functional enzymes.

**Table 1:** Machine learning algorithms that are used for EC number prediction (all methods are accessible through web server)

Method	Feature type	Machine learning algorithm(s)	EC level prediction	Website	Refs.
EzyPred	pseudo PSSM (Pse-PSSM) and FunD encoding	OET-KNN	Three levels	http://www.csbio.sjtu.edu.cn/bioinf/EzyPred/	[53]
SVM-prot	AAC, polarity, hydrophobicity, surface tension, charge, normalized Van der Waals volume, polarizability, secondary structure, solvent accessibility, molecular weight, solubility, number of hydrogen bond donors in side chain, and number of hydrogen bond acceptors in side chain	SVM—KNN—probabilistic neural networks	Three levels	http://jing.cz3.nus.edu.sg/cgi-bin/svmprot.cgi	[54]
DEEPre	sequence length-dependent sequence length independent	CNN—RNN	All levels	http://www.cbrc.kaust.edu.sa/DEEPre	[55]
ECPred	information from amino acid sequence alignment and physicochemical properties	KNN, SVM	All levels	https://ecpred.kansil.org/	[56]
CLEAN	–	Contrastive learning	All levels	https://clean.platform.moleculemaker.org/configuration	[57]
HDMLF	–	Deep learning techniques	All levels	https://ecrecer.biodesign.ac.cn	[58]
EnzBert	–	Transformer model techniques	All levels	https://gitlab.inria.fr/nbuton/tfpc	[102]

##### EzyPred

EzyPred takes a protein sequence as input and then determines whether it is an enzyme. It then proceeds to classify the enzyme into its respective EC number, main EC class, and subclass. The classification of protein sequences in EzyPred is achieved through the implementation of a machine learning approach known as "optimized evidence-theoretic k-nearest neighbor (OET-KNN)" in conjunction with two types of features to capture information about the protein sequence [53].

##### SVM-prot

SVM-prot was initially developed as a computational tool for predicting the EC number of enzymes. It utilizes a representation of the protein sequence using 13 different numerical properties. It employs composition, transition, and distribution to encode each property. The original version used support vector machines (SVM) as the classifier, while it was later updated to utilize two additional classifiers, namely K-nearest neighbors (KNN) and probabilistic neural networks, to expand its prediction capabilities [54]. The incorporation of newer classifiers has significantly improved the overall performance of the method in predicting the EC number of enzymes and their functionality.

##### DEEPre

DEEPre is an EC number prediction tool that employs two types of features for mapping a protein sequence into a numerical space [55]. Sequence length-dependent features, such as position-specific scoring matrices (PSSM), and sequence length-independent features, such as functional domain-based encoding are used as input to a deep learning model comprised of a convolutional neural network (CNN) and recurrent neural network (RNN). DEEPre can predict enzyme function on all four levels of the EC classification system.

##### ECPred

ECPred is another popular method for predicting the EC number of enzymes [56]. This method adopts an independent learning model for each EC number. The classification is carried out in two levels. In the first level, features based on PSSM and physicochemical properties are utilized, and an SVM classifier is employed. In the second level, features derived from sequence alignments are used for classification by a Nearest Neighbor (NN) classifier.

##### CLEAN

CLEAN is a ML algorithm to assign EC number to less-studied proteins or those with uncharacterized functions [57]. CLEAN utilizes a contrastive learning framework, enabling it to confidently assign EC numbers to understudied enzymes, correct mislabeled enzymes, and identify promiscuous enzymes with multiple EC numbers. The effectiveness of CLEAN has been demonstrated through systematic in silico and in vitro experiments.

##### HDMLF

HDMLF is a novel hierarchical dual-core multitask learning framework utilizing advanced deep learning techniques for protein sequence embedding and EC number prediction [58]. An attention layer and a greedy strategy optimize the EC prediction process, resulting in stable and superior performance compared to other representative methods. The tool is accessible through the user-friendly web platform ECRECer (https://ecrecer.biodesign.ac.cn) with a cloud-based serverless architecture and an offline package to enhance usability.

##### EnzBert

EnzBert is a transformer model for sequence-based protein functional annotation [59]. It predicts the functional enzyme annotations by taking into account only sequence features. When compared to state-of-the-art tools, this model demonstrates superior performance in predicting EC numbers. Specifically, the EnzBert model significantly enhanced accuracy in monofunctional enzyme class prediction and achieved a notable improvement in EC number predictions at level two within the benchmark dataset.

#### The integrative approaches based on homology and machine learning

Both HB and MLB approaches can be used to discover novel microbial enzymes from environmental samples. Integrating HB and MLB methods increases the accuracy of enzyme discovery and allows for the targeted mining of novel enzymes, thereby reducing the need for costly and time-consuming wet lab experiments. In a previous study, thermostable xylanases were identified by the HB method and further analyzed using an ML-aided approach based on random forest classification [60]. Specifically, they developed a ML model called TAXyl, based on a SVM, which was trained using various sequence-based and length-independent protein features. The model was designed to discriminate between sequences encoding non-thermophilic, thermophilic, and hyper-thermophilic xylanases. The model was successfully applied to predict three novel thermostable xylanases from sheep and cow rumen metagenomes.

Furthermore, by integrating HB and MLB approaches, the same group also developed an integrated tool called MCIC, which combines HB and MLB analyses to identify cellulases from metagenomic sequences [61]. MCIC focuses on screening novel cellulases based on their optimal pH and temperature dependencies. The machine learning model employed in MCIC was trained using various sequence-based features. The tool facilitates the comparison of metagenome datasets based on their cellulolytic capabilities. To validate the method, two candidate cellulase enzymes identified by MCIC were cloned and subjected to further characterization.

MeTarEnz (metagenomic targeted enzyme miner) (https://cbb.ut.ac.ir/MeTarEnz/) is a similar software providing various services for targeted isolation of different enzymes from user-defined databases. It accepts sequences in different formats including unassembled short reads, assembled contigs, and translated coding sequences. This software can also predict the optimum pH and temperature of lipolytic enzymes using regression models. It was implemented for an in-depth analysis of tannery wastewater metagenomic data followed by mining a thermophilic alkaline lipase [62].

### Utilization of structural information

The primary goal of bioprospecting enzymes for many industrial applications is to identify those that exhibit optimal functionality under specific conditions. Overcoming obstacles and addressing challenges associated with screening methods will contribute to the development of novel tools and technologies for enzyme discovery through metagenomic analysis. By doing so, we can enhance the efficiency and effectiveness of the bioprospecting process, leading to the identification of enzymes with desired characteristics for various industrial applications. Both SBS and FBS methods generate extensive lists of candidate enzymes. However, characterizing these candidates and identifying specific enzymes with desired properties remains a challenging task. Structural analyses can play a crucial role in narrowing down the search space by reducing the candidate sequences to a limited subset. This targeted subset can then undergo further functional analysis through wet lab procedures. By integrating structural analyses, researchers can efficiently prioritize and focus their experimental efforts on a more manageable set of candidate enzymes, facilitating the identification of enzymes with the desired properties.

It is widely accepted that the 3D structure of an enzyme directly influences its function. However, there are also instances where proteins with similar sequences exhibit dissimilar structures [63]. Surprisingly, even highly similar sequences can lead to proteins with distinct structures. This observed structural dissimilarity often correlates with differences in their functions [63]. There are also examples of proteins with limited sequence similarities but the same folding structures, suggesting that conserved positions in proteins tend to preserve their folding and biological functionality [64]. These findings highlight the complex relationship between protein sequence, structure, and function, demonstrating that sequence similarity alone cannot reliably predict structural similarities or functional properties of enzymes.

The analysis of protein structure–function relationships can be conducted at three levels: amino acid sequence and composition, 3D structure, and spatial conformations of the active site [65]. Computational molecular simulation offers a robust approach for determining and analyzing enzyme structure, dynamics, and functional mechanisms within the framework of physical interactions. Analyzing the 3D structures of enzymes can provide valuable insights into their diverse properties, such as function, spatial conformation, thermal and pH stability.

Prominent methods for predicting 3D protein structures include comparative modeling and ab initio structure prediction [66]. Comparative modeling can be achieved through homology modeling or threading methods for fold recognition. In homology modeling, predictions are based on previously solved structures serving as templates, assuming that homologous proteins share similar 3D structures. Choosing an appropriate template model is crucial for achieving high-quality and accurate predictions. Threading methods involve scanning the primary structure of an unknown protein against a database of proteins with known structures [67, 68]. By employing scoring functions based on statistical or knowledge-based potentials, the compatibility of the query protein with known structure is evaluated. Commonly used tools for comparative modeling include I-TASSER [69], Phyre [70], MODELLER [71], SWISS-MODEL [72], and AlphaFold [73]. Particularly, AlphaFold represents a significant advancement in structure prediction methodologies, leveraging state-of-the-art neural network architectures and training procedures. By integrating evolutionary, physical, and geometric constraints specific to protein structures, AlphaFold achieves remarkable improvements in accuracy.

Ab initio protein structure modeling involves the prediction of protein structures from scratch, relying solely on physical forces and energy principles [74]. This approach is particularly valuable when experimental structural information or suitable template structures are unavailable. Various tools are available to perform ab initio structural prediction, each utilizing different algorithms and methodologies. Notable examples include GROMACS [75], NAMD [76], and TeraChem [77]. These tools employ advanced simulation techniques such as molecular dynamics to explore the conformational space and identify the most energetically favorable protein structure. By leveraging the principles of physics and energy minimization, ab initio modeling enables the generation of protein structures in the absence of prior structural knowledge.

Protein 3D structure modeling plays a crucial role in distinguishing proteins with similar sequences, allowing the exploration of hidden characteristics that cannot be revealed through conventional sequence homology searches alone. This capability becomes particularly valuable when searching for novel enzymes within protein sequences predicted from metagenome data. By providing detailed insights into the spatial arrangement of atoms within a protein, 3D structure modeling aids in the identification of unique structural features, functional regions, and key residues that contribute to enzyme activity. This deeper understanding of protein structure allows for more precise and comprehensive analysis, ultimately facilitating the discovery and characterization of novel enzymes with desired properties from metagenome-derived sequences.

The utilization of structural information has been extensively employed in enzyme bioprospecting from environmental samples, as demonstrated by various studies summarized in Table 2. The processes that lead to the identification of candidate enzymes are summarized into seven distinct stages (S1-S6), with each stage involving specific computational analyses. The different stages of enzyme bioprospecting and their corresponding computational analyses are presented in Table 2.

**Table 2:** The list of candidate enzymes discovered through integrated sequence and structure analyses

Novel metagenomic enzyme	Environment	Computational multi-stage pipeline						
		S1	S2	S3	S4	S5	S6	Refs.
Alkali-thermostable xylanases	*Aspergillus fumigatus*	✓	✓	✓	✓	✓	✓	[103]
Xylanase (XynNTU)	*Paenibacillus campinasensis NTU-11*	–	✓	✓	–	–	–	[104]
Extreme halophilic xylanase	Camel rumen microbiome	✓	–	✓	–	✓	✓	[105]
Thermostable xylanase	Hot spring microbiome	✓	✓	✓	–	✓	✓	[106]
Alkali-thermostable xylanase	Termite gut microbiome	✓	✓	✓	–	–	✓	[107]
Thermostable xylanase	Hot sediment microbiome	✓	✓	✓	–	–	–	[108]
Thermostable xylanase	Hot spring sediment microbiome	✓	✓	✓	–	–	✓	[109]
Thermostable xylanase	Camel rumen microbiome	✓	–	–	–	–	✓	[110]
Thermostable xylanase	Cattle rumen microbiome	–	✓	✓	✓	–	✓	[111]
Thermostable xylanase	Pulp and paper wastewater microbiome	–	✓	✓	✓	–	–	[112]
Alkali-thermostable xylanases *(PersiXyn1)*	Camel rumen microbiome	✓	–	✓	✓	–	✓	[113]
Alkali-thermostable xylanases *(PersiXyn2)*	Camel rumen microbiome	✓	✓	✓	✓	–	✓	[114]
Alkali-thermostable Xylanase *(PersiXyn3,4)*	Cattle rumen microbiome	✓	–	✓	✓	✓	✓	[115]
Thermal dependent xylanases *(PersiXyn5,6,7)*	Sheep and cattle rumen microbiome	✓	–	✓	✓	✓	✓	[60]
Thermostable xylanase *(PersiXyn8)*	Cattle rumen microbiome	✓	–	✓	✓	✓	✓	[116]
Hyperthermostable xylanase *(PersiXyn10)*	Camel rumen microbiome	✓	–	✓	✓	–	✓	[117]
xylanase/ esterase	Cattle rumen microbiome	✓	–	✓	✓	–	–	[118]
Bifunctional mannanase/xylanase *(PersiManXyn1)*	Sheep rumen microbiome	✓	–	✓	✓	–	✓	[119]
Xylanase/β-glucosidase *(PersiBGLXyn1)*	Cattle rumen microbiome	✓	–	✓	✓	–	✓	[120]
Thermostable cellulase	Soil microbiome	–	✓	✓	✓	✓	✓	[121]
Thermostable cellulase	Cattle rumen microbiome	✓	–	✓	✓	–	✓	[122]
Hyperthermophilic cellulase	Arctic Mid-Ocean Ridge vent field microbiome	✓	–	✓	✓	–	✓	[123]
Acidic cellulase	Buffalo rumen microbiome	✓	✓	✓	✓	–	✓	[124]
Alkaline-thermostable cellulase	Goat rumen microbiome	✓	–	✓	✓	–	✓	[125]
Thermostable endoglucanase	Termite gut microbiome	✓	✓	✓	✓	–	✓	[126]
Alkalic and thermostable cellulase *(PersiCel1,2)*	Camel rumen microbiome	✓	–	✓	✓	✓	✓	[127]
Thermostable and halotolerant cellulase *(PersiCel3)*	Sheep rumen microbiome	✓	–	✓	✓	✓	✓	[128]
Alkali-thermostable endo-β-1,4-glucanase *(PersiCel4)*	Sheep rumen microbiome	✓	✓	✓	✓	✓	✓	[129]
Cellulase/Hemicellulase	Soil microbiome	✓	✓	✓	✓	✓	–	[130]
Alkalophilic, thermophilic carboxylesterase	Soil microbiome	✓	✓	✓	✓	–	–	[131]
Carboxylesterase	Soil microbiome	✓	✓	✓	✓	–	–	[132]
Carboxylesterase	Compost microbiome	✓	✓	✓	✓	–	✓	[133]
Carboxylesterase	Sediment microbiome	✓	✓	✓	✓	–	–	[33]
Thermostable bifunctional cellulase/xylanase *(PersiCelXyn1)*	Cattle rumen microbiome	✓	–	✓	✓	✓	✓	[134]
Glucose and ethanol tolerant β-Glucosidase	Hot spring microbiome	✓	✓	✓	✓	✓	✓	[135]
β-glucosidase, α-L-arabinofuranosidase, β-xylosidase, and endo-1,4-β-xylanase	Porcupine microbiome	✓	✓	✓	✓	✓	–	[136]
Homologue of human α-glucosidase *(PersiAlpha-GL1)*	In vitro gastrointestinal digestion	✓	–	✓	✓	✓	✓	[137]
α-amylase *(PersiAmy1)*	Sheep rumen microbiome	✓	–	✓	✓	✓	✓	[138]
Acidic-thermostable α-amylase *(PersiAmy2)*	Sheep rumen microbiome	✓	–	✓	✓	✓	✓	[139]
Acidic-Thermostable $\alpha$-amylase *(PersiAmy3)*	Sheep rumen microbiome	✓	✓	✓	✓	✓	✓	[139]
Cold-active pullulanase	Hot spring microbiome	✓	✓	✓	✓	–	✓	[140]
Thermostable pullulanase *(PersiPul1)*	Cattle rumen microbiome	✓	–	✓	✓	✓	✓	[141]
Laccase	Soil microbiome	✓	–	✓	✓	–	–	[142]
Stable laccase *(PersiLac1)*	Tannery wastewater microbiome	✓	–	✓	✓	✓	✓	[143]
Thermo-halotolerant laccase *(PersiLac2)*	Tannery wastewater microbiome	✓	–	✓	✓	✓	✓	[144]
Protease	Solid tannery waste microbiome	✓	✓	✓	✓	–	–	[145]
Protease	Solid tannery waste microbiome	✓	✓	✓	✓	–	✓	[146]
Thermo-halo-alkali-stable protease *(PersiProtease1)*	Tannery wastewater microbiome	✓	–	✓	✓	✓	✓	[147]
Feruloyl esterase	Soil microbiome	✓	✓	✓	✓	–	–	[148]
Solvent-tolerant esterase	Compost microbiome	✓	✓	✓	✓	–	✓	[35]
Esterase	Wastewater sediments microbiome	✓	✓	✓	✓	–	–	[149]
Esterase	Soil microbiome	✓	✓	✓	✓	–	✓	[150]
Lipid hydrolyzing enzyme	Hot spring microbiome	✓	–	✓	✓	–	✓	[151]
Tyrosine Phosphatase	Soil microbiome	✓	✓	✓	✓	✓	✓	[152]
PETase	Environmental metagenome	✓	✓	✓	✓	–	✓	[153]
PETase	Human saliva microbiome	✓	✓	✓	✓	✓	–	[154]
β-galactosidase	Marine microbiome	✓	✓	✓	✓	–	–	[155]
β -Glucuronidase	Mouse gut microbiome	✓	✓	✓	✓	–	–	[156]
β -Glucanase	Soil microbiome	✓	✓	✓	✓	–	✓	[157]
β -Glucanase	Vermicompost	✓	✓	✓	✓	–	–	[158]
ferulic acid esterase, α-L-arabinofuranosidase, GH10 β-D-1,4-xylanase	Wastewater treatment sludge	✓	–	–	–	–	–	[159]

The table includes information about the enzyme family, metagenome source, and the *in-silico* analyses conducted during the bioprospecting processes. Detailed analysis steps are outlined below

S1: BLAST alignment of metagenome sequences against a curated list of experimentally validated enzymes with desired properties obtained from a literature review. Selection of the most similar sequences, determined by their E-value and alignment score, for further refinement

S2: Analysis of the selected sequences to determine their phylogenetic positions among the related sequences obtained from the literature search. Focus on closely related sequences after removing distant relatives

S3: Determining the frequency and position of important amino acids in the candidate metagenome sequences. Assessment of statistical compatibility of these amino acids with literature and experimentally characterized enzymes possessing desired properties. This stage requires in-depth and comprehensive review of literature

S4: Comparing the candidate sequences for their active sites and other key amino acids with enzymes possessing the desired properties

S5: At this stage, the presence of conserved domains in the candidate enzymes is confirmed by utilizing tools such as CDD [141], Position Specific Scoring Matrices (PSSMs) or Hidden Markov Models (HMMs) or other motif modeling strategies

S6: Predicting the 3D structure of the candidate sequences and filtering for less related sequences

#### Predicting enzyme thermal stability through structural analysis

The 3D structure of native proteins is determined by a multitude of weak interactions, including hydrogen bonding, salt bridges, hydrophobic, and polar interactions. These non-covalent forces, along with covalent disulfide bonds between cysteine residues, play essential roles in stabilizing protein structure [78]. These interactions contribute to various structural properties such as protein stability, dynamics, recognition, catalysis, and degradation. Salt bridges are strong electrostatic interactions formed between negatively charged groups [79] that stabilize protein structure and protect the protein from aggregation [80]. The stability of salt bridges is influenced by factors such as pH, distance and geometric orientation of the residues involved. Predicting the presence and location of salt bridges in a protein provides valuable insight into protein stability. There are several freely available tools to predict salt bridges, including Tm predictor [http://tm.life.nthu.edu.tw/], PoPMusic [81], and SCooP [82]. These tools are mainly used to predict changes in the thermodynamic stability, melting temperature, and temperature-dependent stability of a protein.

Hydrogen bonds are another crucial type of interaction that contributes to protein structure. They play a key role in the formation of secondary structures, such as $\mathrm{\alpha }$-helices and $\upbeta$-sheets, by establishing bonds between carbonyl oxygen and amide nitrogen [83]. Several tools are available for predicting the number of hydrogen bonds in a protein, including HBPLUS [84], PyMol [85], and HAAD [86].

Disulfide bonds also play a vital role in the formation of protein structures. They contribute to the stability of protein structures under harsh environments, enhance their mechanical and thermodynamic stability, and minimize the likelihood of misfolding [87]. Computational tools have been developed to accurately predict disulfide-bonding networks and patterns in a protein, thereby aiding in the correct modeling of protein structure. Fariselli et al. [78] introduced a tool for predicting the disulfide bonding state of cysteines in proteins with a prediction accuracy of over 90%.

## Natural product discovery through metagenomics

Traditionally, the search for bioactive natural products in microorganisms relied largely on activity-based screening approaches [88], which in turn necessitate the isolation and pure culture of the source microorganism. However, recent advances in culture-independent metagenomic and bioinformatic analyses have made it possible to search for novel natural products in microorganisms without the need for their pure culture. This approach offers the exciting potential to delve into the enzymatic mechanisms involved in the biosynthesis and modification of these natural medicinally important compounds. Despite the structural complexity of natural products, their biosynthetic pathways and the enzymes involved in their bioconversion exhibit a remarkable degree of conservation across diverse microbial lineages [23]. This conservation facilitates the discovery, annotation, and characterization of novel natural product biosynthetic enzymes and pathways through sequence homology searches and structural predictions [89]. The combined application of advanced bioinformatic tools and high-throughput screening methodologies offers a powerful approach for targeted mining of metagenomic data, with the potential to significantly accelerate the discovery of novel natural product biosynthesis pathways and subsequent characterization of valuable therapeutic agents and bioactive compounds.

The majority of bacterial natural products fall into the category of secondary metabolites that are encoded by conserved biosynthetic gene clusters (BGCs), a group of two or more closely linked genes that encode enzymes of the biosynthetic pathway for a specific metabolite or natural product [90]. This genomic organization facilitates the identification of natural products through genome mining approaches. Genome mining tools such as antiSMASH [91], PRISM [92], CLUSEAN [93], NP.searcher [94], and NRPminer [95] have been developed to identify putative BGCs in genome or metagenome datasets. AntiSMASH stands out among other tools by offering a comprehensive suite of tools and databases for automated genome mining of a wide array of secondary metabolites. By combining genome mining for BGCs and chemical structure prediction for the encoded secondary metabolites, PRISM significantly improves the detection of genetically encoded nonribosomal peptides and polyketides [92]. While these tools facilitate the identification of genomic loci responsible for natural product biosynthesis, challenges arise in connecting these loci to the specific chemical structures of the encoded products [96]. Genomic analysis has revealed that bacterial genomes house numerous orphan BGCs, which are clusters not yet associated with the natural products they encode. There are also numerous examples of isolated natural products that have not been linked to their corresponding BGCs [97]. ML approaches have shown potential in genome mining for natural biological products, predicting the structure of natural products, and inferring biological activity from BGCs or the chemical structure of the respective secondary metabolite. Recently Prihoda et al. [98] showed that ML can be used in several steps to find bioactive natural products in genome sequences, including genome annotations, feature representation, BGC detection, structure prediction, and activity profiling. Another study developed a comprehensive ML method to predict the structures and biological activity of secondary metabolites from microbial genome sequences [99]. This approach can be used to predict the structures of natural products encoded by orphan BGCs.

In light of widespread metagenomic explorations of diverse microbial niches, huge amounts of genomic data are now at our fingertips. This genetic bounty holds immense potential for bioprospecting, offering novel microbial secondary metabolites, with a spectrum of promising medical and biotechnological applications. Numerous attempts have been made to explore metagenome data for novel natural products. In a study by Nayfach et al. [9], over 100,000 BGCs were predicted in 52,515 metagenome-assembled genomes, which were cataloged from diverse microbial communities representing the Earth's microbiome. This antiSMASH-based BGC discovery yielded up to 54 times more BGCs than manually curated entries in the MIBiG dataset, highlighting a vast reservoir of unexplored microbial natural products. In a comprehensive computational and experimental study, a probabilistic algorithm named MetaBGC was developed and applied to identify potential BGCs in complex metagenomic sequences from various regions of the human microbiome (gut, mouth, skin, and vagina) [100]. Out of the 13 BGCs encoding type II polyketides, two were successfully cloned and expressed in a heterologous system, revealing their potent antibacterial activities against gut microbes and suggesting a potential role in microbial interactions within the gut environment. These findings underscore the urgent need for the development of advanced tools and pipelines for targeted mining of metagenomes for novel, game-changing microbial secondary metabolites with biotechnological and medicinal potential.

## Future directions

An extensive literature review highlights that functional screening is, in fact, a major source of currently characterized enzymes from environmental samples. However, there are instances where the integration of FBS and SBS methods has proven to be successful. For example, in a study on the pre-screening of clone libraries using functional screening followed by insert sequencing, a remarkable 106-fold increase in the success rate was achieved in identifying genes encoding desired enzymes compared to direct sequencing approaches [101]. Both FBS and SBS methods offer distinct advantages and disadvantages. SBS approaches may have limitations in terms of sequencing cost and errors. Furthermore, uncertainty in functional annotations and their limitations in discovering novel enzymes pose challenges to their widespread applications. FBS approaches can be used to identify novel enzymes and facilitate the direct determination of gene functions. However, the FBS methods also suffer from higher costs, the lack of effective screening methods for certain enzyme activities, and the challenges associated with heterologous expression systems.

In the past decade, significant improvements have been made in the computational modeling of 3D structures of proteins. These advancements have made it possible to take advantage of protein structure modeling in screening for novel enzymes from metagenomic sequences. Structural modeling can be used to evaluate enzymes for substrate specificity, enantioselectivity, metal ion specificity, pH and temperature dependence, as well as stability and secondary catalytic function.

In the era of a rapid expansion in enzyme-related biological databases as repositories for genome sequences, enzymes, tertiary structures, active sites, as well as metabolic pathways and reactions, there is an increased demand for the development of functional and computational screening tools. It is evident that the integration of SBS and FBS methods, coupled with the utilization of structural modeling, paves the way toward efficient exploration of novel enzymes from high throughput metagenomic data. This combination of approaches presents a promising roadmap for effective enzyme and natural product mining in the future.

## References

[1] Gurung N, Ray S, Bose S, Rai V (2013). A broader view: microbial enzymes and their relevance in industries, medicine, and beyond. Biomed Res Int.

[2] Guazzaroni ME, Beloqui A, Vieites JM, Al-ramahi Y, Cortés NL, Ghazi A, et al. Metagenomic mining of enzyme diversity. Handbook of hydrocarbon and lipid microbiology. 2010. p. 2911–27.

[3] Liu X, Kokare C. Microbial enzymes of use in industry. Biotechnology of microbial enzymes. 2017. p. 267–98.

[4] Singh RS, Singh T, Pandey A. microbial enzymes—an overview. Advances in Enzyme Technology. 2019. p. 1–40.

[5] Lammle K, Zipper H, Breuer M, Hauer B, Buta C, Brunner H (2007). Identification of novel enzymes with different hydrolytic activities by metagenome expression cloning. J Biotechnol.

[6] Amann RI, Binder BJ, Olson RJ, Chisholm SW, Devereux R, Stahl DA (1990). Combination of 16S rRNA-targeted oligonucleotide probes with flow cytometry for analyzing mixed microbial populations. Appl Environ Microbiol.

[7] Glogauer A, Martini VP, Faoro H, Couto GH, Muller-Santos M, Monteiro RA (2011). Identification and characterization of a new true lipase isolated through metagenomic approach. Microb Cell Fact.

[8] Quince C, Walker AW, Simpson JT, Loman NJ, Segata N (2017). Corrigendum: shotgun metagenomics, from sampling to analysis. Nat Biotechnol.

[9] Nayfach S, Roux S, Seshadri R, Udwary D, Varghese N, Schulz F (2021). A genomic catalog of Earth's microbiomes. Nat Biotechnol.

[10] Berini F, Casciello C, Marcone GL, Marinelli F (2017). Metagenomics: novel enzymes from non-culturable microbes. FEMS Microbiol Lett..

[11] Itoh N. Metagenomics for improved biocatalysis. Future directions in biocatalysis. 2017. p. 375–84.

[12] Colin PY, Kintses B, Gielen F, Miton CM, Fischer G, Mohamed MF (2015). Ultrahigh-throughput discovery of promiscuous enzymes by picodroplet functional metagenomics. Nat Commun.

[13] Arnold FH (2001). Combinatorial and computational challenges for biocatalyst design. Nature.

[14] Robinson SL, Piel J, Sunagawa S (2021). A roadmap for metagenomic enzyme discovery. Nat Prod Rep.

[15] Hou Q, Pucci F, Pan F, Xue F, Rooman M, Feng Q (2022). Using metagenomic data to boost protein structure prediction and discovery. Comput Struct Biotechnol J.

[16] Jeske L, Placzek S, Schomburg I, Chang A, Schomburg D (2019). BRENDA in 2019: a European ELIXIR core data resource. Nucleic Acids Res.

[17] Atkinson HJ, Morris JH, Ferrin TE, Babbitt PC (2009). Using sequence similarity networks for visualization of relationships across diverse protein superfamilies. PLoS ONE.

[18] Lapebie P, Lombard V, Drula E, Terrapon N, Henrissat B (2019). Bacteroidetes use thousands of enzyme combinations to break down glycans. Nat Commun.

[19] Gharechahi J, Vahidi MF, Sharifi G, Ariaeenejad S, Ding XZ, Han JL (2023). Lignocellulose degradation by rumen bacterial communities: new insights from metagenome analyses. Environ Res.

[20] Ngara TR, Zhang H (2018). Recent advances in function-based metagenomic screening. Genom Proteom Bioinform.

[21] Patel T, Chaudhari HG, Prajapati V, Patel S, Mehta V, Soni N (2022). A brief account on enzyme mining using metagenomic approach. Front Syst Biol..

[22] Sampaio PS, Fernandes P (2023). Machine learning: a suitable method for biocatalysis. Catalysts.

[23] Scherlach K, Hertweck C (2021). Mining and unearthing hidden biosynthetic potential. Nat Commun..

[24] Zaparucha A, de Berardinis V, Vaxelaire-Vergne C. Chapter 1. Genome Mining for Enzyme Discovery. Modern Biocatalysis. Catalysis Series, 2018. p. 1–27.

[25] Staley JT, Konopka A (1985). Measurement of in situ activities of nonphotosynthetic microorganisms in aquatic and terrestrial habitats. Annu Rev Microbiol.

[26] Uchiyama T, Miyazaki K (2009). Functional metagenomics for enzyme discovery: challenges to efficient screening. Curr Opin Biotechnol.

[27] Daniel R (2004). The soil metagenome–a rich resource for the discovery of novel natural products. Curr Opin Biotechnol.

[28] Yun J, Ryu S (2005). Screening for novel enzymes from metagenome and SIGEX, as a way to improve it. Microb Cell Fact.

[29] Madhavan A, Sindhu R, Parameswaran B, Sukumaran RK, Pandey A (2017). Metagenome analysis: a powerful tool for enzyme bioprospecting. Appl Biochem Biotechnol.

[30] Dadheech T, Shah R, Pandit R, Hinsu A, Chauhan PS, Jakhesara S (2018). Cloning, molecular modeling and characterization of acidic cellulase from buffalo rumen and its applicability in saccharification of lignocellulosic biomass. Int J Biol Macromol.

[31] De Santi C, Altermark B, Pierechod MM, Ambrosino L, de Pascale D, Willassen NP (2016). Characterization of a cold-active and salt tolerant esterase identified by functional screening of Arctic metagenomic libraries. BMC Biochem.

[32] Pereira MR, Maester TC, Mercaldi GF, de Macedo Lemos EG, Hyvonen M, Balan A (2017). From a metagenomic source to a high-resolution structure of a novel alkaline esterase. Appl Microbiol Biotechnol.

[33] Araujo FJ, Hissa DC, Silva GO, Antunes A, Nogueira VLR, Goncalves LRB (2020). A novel bacterial carboxylesterase identified in a metagenome derived-clone from Brazilian mangrove sediments. Mol Biol Rep.

[34] Istvan P, Souza AA, Garay AV, Dos Santos DFK, de Oliveira GM, Santana RH (2018). Structural and functional characterization of a novel lipolytic enzyme from a Brazilian Cerrado soil metagenomic library. Biotechnol Lett.

[35] Park JM, Kang CH, Won SM, Oh KH, Yoon JH (2019). Characterization of a novel moderately thermophilic solvent-tolerant esterase isolated from a compost metagenome library. Front Microbiol.

[36] Maruthamuthu M, van Elsas JD (2017). Molecular cloning, expression, and characterization of four novel thermo-alkaliphilic enzymes retrieved from a metagenomic library. Biotechnol Biofuels.

[37] Thomas T, Gilbert J, Meyer F (2012). Metagenomics - a guide from sampling to data analysis. Microb Inform Exp.

[38] Ovchinnikov S, Park H, Varghese N, Huang PS, Pavlopoulos GA, Kim DE (2017). Protein structure determination using metagenome sequence data. Science.

[39] Wang Y, Shi Q, Yang P, Zhang C, Mortuza SM, Xue Z (2019). Fueling ab initio folding with marine metagenomics enables structure and function predictions of new protein families. Genome Biol.

[40] Altschul SF, Gish W, Miller W, Myers EW, Lipman DJ (1990). Basic local alignment search tool. J Mol Biol.

[41] Buchfink B, Xie C, Huson DH (2015). Fast and sensitive protein alignment using DIAMOND. Nat Methods.

[42] Edgar RC (2010). Search and clustering orders of magnitude faster than BLAST. Bioinformatics.

[43] Eddy SR (1998). Profile hidden Markov models. Bioinformatics.

[44] Altschul SF, Madden TL, Schaffer AA, Zhang J, Zhang Z, Miller W (1997). Gapped BLAST and PSI-BLAST: a new generation of protein database search programs. Nucleic Acids Res.

[45] Szalkai B, Grolmusz V (2019). MetaHMM: a webserver for identifying novel genes with specified functions in metagenomic samples. Genomics.

[46] Koutsandreas T, Ladoukakis E, Pilalis E, Zarafeta D, Kolisis FN, Skretas G (2019). ANASTASIA: an automated metagenomic analysis pipeline for novel enzyme discovery exploiting next generation sequencing data. Front Genet.

[47] Nobeli I, Favia AD, Thornton JM (2009). Protein promiscuity and its implications for biotechnology. Nat Biotechnol.

[48] Elbehery AH, Leak DJ, Siam R (2017). Novel thermostable antibiotic resistance enzymes from the Atlantis II Deep Red Sea brine pool. Microb Biotechnol.

[49] Garg R, Srivastava R, Brahma V, Verma L, Karthikeyan S, Sahni G (2016). Biochemical and structural characterization of a novel halotolerant cellulase from soil metagenome. Sci Rep.

[50] Al-Shahib A, Breitling R, Gilbert DR (2007). Predicting protein function by machine learning on amino acid sequences–a critical evaluation. BMC Genomics.

[51] Bonetta R, Valentino G (2020). Machine learning techniques for protein function prediction. Proteins.

[52] Zou Z, Tian S, Gao X, Li Y (2018). mlDEEPre: multi-functional enzyme function prediction with hierarchical multi-label deep learning. Front Genet.

[53] Shen HB, Chou KC (2007). EzyPred: a top-down approach for predicting enzyme functional classes and subclasses. Biochem Biophys Res Commun.

[54] Li YH, Xu JY, Tao L, Li XF, Li S, Zeng X (2016). SVM-Prot 2016: a web-server for machine learning prediction of protein functional families from sequence irrespective of similarity. PLoS ONE.

[55] Li Y, Wang S, Umarov R, Xie B, Fan M, Li L (2018). DEEPre: sequence-based enzyme EC number prediction by deep learning. Bioinformatics.

[56] Dalkiran A, Rifaioglu AS, Martin MJ, Cetin-Atalay R, Atalay V, Dogan T (2018). ECPred: a tool for the prediction of the enzymatic functions of protein sequences based on the EC nomenclature. BMC Bioinformatics.

[57] Yu T, Cui H, Li JC, Luo Y, Jiang G, Zhao H (2023). Enzyme function prediction using contrastive learning. Science.

[58] Shi Z, Deng R, Yuan Q, Mao Z, Wang R, Li H (2023). Enzyme commission number prediction and benchmarking with hierarchical dual-core multitask learning framework. Research.

[59] Buton N. Datasets and models for EnzBert. Zenodo; 2023.

[60] Foroozandeh Shahraki M, Farhadyar K, Kavousi K, Azarabad MH, Boroomand A, Ariaeenejad S (2021). A generalized machine-learning aided method for targeted identification of industrial enzymes from metagenome: a xylanase temperature dependence case study. Biotechnol Bioeng.

[61] Foroozandeh Shahraki M, Ariaeenejad S, Fallah Atanaki F, Zolfaghari B, Koshiba T, Kavousi K (2020). MCIC: automated identification of cellulases from metagenomic data and characterization based on temperature and pH dependence. Front Microbiol.

[62] Shahraki MF, Atanaki FF, Ariaeenejad S, Ghaffari MR, Norouzi-Beirami MH, Maleki M (2022). A computational learning paradigm to targeted discovery of biocatalysts from metagenomic data: a case study of lipase identification. Biotechnol Bioeng.

[63] Kosloff M, Kolodny R (2008). Sequence-similar, structure-dissimilar protein pairs in the PDB. Proteins.

[64] Friedberg I, Margalit H (2002). Persistently conserved positions in structurally similar, sequence dissimilar proteins: roles in preserving protein fold and function. Protein Sci.

[65] Littlechild JA. Protein structure and function. Introduction to biological and small molecule drug research and development. 2013. p. 57–79.

[66] Petrey D, Honig B (2005). Protein structure prediction: inroads to biology. Mol Cell.

[67] Madej T, Gibrat JF, Bryant SH (1995). Threading a database of protein cores. Proteins.

[68] Bertoline LMF, Lima AN, Krieger JE, Teixeira SK (2023). Before and after AlphaFold2: an overview of protein structure prediction. Front Bioinform.

[69] Yang J, Yan R, Roy A, Xu D, Poisson J, Zhang Y (2015). The I-TASSER Suite: protein structure and function prediction. Nat Methods.

[70] Kelley LA, Mezulis S, Yates CM, Wass MN, Sternberg MJ (2015). The Phyre2 web portal for protein modeling, prediction and analysis. Nat Protoc.

[71] Webb B, Sali A (2016). Comparative protein structure modeling using modeller. Curr Protoc Bioinformatics..

[72] Biasini M, Bienert S, Waterhouse A, Arnold K, Studer G, Schmidt T, et al. SWISS-MODEL: modelling protein tertiary and quaternary structure using evolutionary information. Nucleic Acids Res. 2014;42(Web Server issue):W252–8. 10.1093/nar/gku340. 10.1093/nar/gku340 PMC4086089 24782522

[73] Jumper J, Evans R, Pritzel A, Green T, Figurnov M, Ronneberger O (2021). Highly accurate protein structure prediction with AlphaFold. Nature.

[74] Baltzer L, Nilsson H, Nilsson J (2001). De novo design of proteins–what are the rules?. Chem Rev.

[75] Berendsen HJC, van der Spoel D, van Drunen R (1995). GROMACS: a message-passing parallel molecular dynamics implementation. Comput Phys Commun.

[76] Nelson MT, Humphrey W, Gursoy A, Dalke A, Kalé LV, Skeel RD (2016). NAMD: a parallel, object-oriented molecular dynamics program. Int J High Perform Comput Appl.

[77] Seritan S, Bannwarth C, Fales BS, Hohenstein EG, Isborn CM, Kokkila-Schumacher SIL (2020). TeraChem: a graphical processing unit-accelerated electronic structure package for large-scale ab initio molecular dynamics. WIREs Comput Mol Sci..

[78] Fariselli P, Riccobelli P, Casadio R (1999). Role of evolutionary information in predicting the disulfide-bonding state of cysteine in proteins. Proteins.

[79] Ban X, Lahiri P, Dhoble AS, Li D, Gu Z, Li C (2019). Evolutionary stability of salt bridges hints its contribution to stability of proteins. Comput Struct Biotechnol J.

[80] Ahmed MC, Papaleo E, Lindorff-Larsen K (2018). How well do force fields capture the strength of salt bridges in proteins?. PeerJ.

[81] Dehouck Y, Kwasigroch JM, Gilis D, Rooman M (2011). PoPMuSiC 2.1: a web server for the estimation of protein stability changes upon mutation and sequence optimality. BMC Bioinformatics.

[82] Pucci F, Kwasigroch JM, Rooman M (2017). SCooP: an accurate and fast predictor of protein stability curves as a function of temperature. Bioinformatics.

[83] Hubbard RE, Kamran Haider M. Hydrogen bonds in proteins: role and strength. eLS. 2010.

[84] McDonald IK, Thornton JM (1994). Satisfying hydrogen bonding potential in proteins. J Mol Biol.

[85] odinger L. The PyMOL molecular graphics system, version 2.0 Schrödinger, LLC. 2015.

[86] Li Y, Roy A, Zhang Y (2009). HAAD: A quick algorithm for accurate prediction of hydrogen atoms in protein structures. PLoS ONE.

[87] Salam NK, Adzhigirey M, Sherman W, Pearlman DA (2014). Structure-based approach to the prediction of disulfide bonds in proteins. Protein Eng Des Sel.

[88] Katz L, Baltz RH (2016). Natural product discovery: past, present, and future. J Ind Microbiol Biotechnol.

[89] Scott TA, Piel J (2019). The hidden enzymology of bacterial natural product biosynthesis. Nat Rev Chem.

[90] Medema MH, Kottmann R, Yilmaz P, Cummings M, Biggins JB, Blin K (2015). Minimum information about a biosynthetic gene cluster. Nat Chem Biol.

[91] Blin K, Shaw S, Augustijn HE, Reitz ZL, Biermann F, Alanjary M (2023). antiSMASH 7.0: new and improved predictions for detection, regulation, chemical structures and visualisation. Nucleic Acids Res.

[92] Skinnider MA, Merwin NJ, Johnston CW, Magarvey NA (2017). PRISM 3: expanded prediction of natural product chemical structures from microbial genomes. Nucleic Acids Res.

[93] Weber T, Rausch C, Lopez P, Hoof I, Gaykova V, Huson DH (2009). CLUSEAN: a computer-based framework for the automated analysis of bacterial secondary metabolite biosynthetic gene clusters. J Biotechnol.

[94] Li MH, Ung PM, Zajkowski J, Garneau-Tsodikova S, Sherman DH (2009). Automated genome mining for natural products. BMC Bioinformatics.

[95] Behsaz B, Bode E, Gurevich A, Shi YN, Grundmann F, Acharya D (2021). Integrating genomics and metabolomics for scalable non-ribosomal peptide discovery. Nat Commun.

[96] Skinnider MA, Johnston CW, Gunabalasingam M, Merwin NJ, Kieliszek AM, MacLellan RJ (2020). Comprehensive prediction of secondary metabolite structure and biological activity from microbial genome sequences. Nat Commun.

[97] Jensen PR (2016). Natural products and the gene cluster revolution. Trends Microbiol.

[98] Prihoda D, Maritz JM, Klempir O, Dzamba D, Woelk CH, Hazuda DJ (2021). The application potential of machine learning and genomics for understanding natural product diversity, chemistry, and therapeutic translatability. Nat Prod Rep.

[99] Walker AS, Clardy J (2021). A machine learning bioinformatics method to predict biological activity from biosynthetic gene clusters. J Chem Inf Model.

[100] Sugimoto Y, Camacho FR, Wang S, Chankhamjon P, Odabas A, Biswas A (2019). A metagenomic strategy for harnessing the chemical repertoire of the human microbiome. Science.

[101] Tasse L, Bercovici J, Pizzut-Serin S, Robe P, Tap J, Klopp C (2010). Functional metagenomics to mine the human gut microbiome for dietary fiber catabolic enzymes. Genome Res.

[102] Buton N, Coste F, Le Cunff Y, Valencia A (2023). Predicting enzymatic function of protein sequences with attention. Bioinformatics.

[103] Dodda SR, Hossain M, Kapoor BS, Dasgupta S, Aikat K (2021). Computational approach for identification, characterization, three-dimensional structure modelling and machine learning-based thermostability prediction of xylanases from the genome of *Aspergillus fumigatus*. Comput Biol Chem..

[104] Wang L, Wang Y, Chang S, Gao Z, Ma J, Wu B (2022). Identification and characterization of a thermostable GH11 xylanase from Paenibacillus campinasensis NTU-11 and the distinct roles of its carbohydrate-binding domain and linker sequence. Colloids Surf B Biointerfaces.

[105] Ghadikolaei KK, Sangachini ED, Vahdatirad V, Noghabi KA, Zahiri HS (2019). An extreme halophilic xylanase from camel rumen metagenome with elevated catalytic activity in high salt concentrations. AMB Express.

[106] Joshi N, Sharma M, Singh SP (2020). Characterization of a novel xylanase from an extreme temperature hot spring metagenome for xylooligosaccharide production. Appl Microbiol Biotechnol.

[107] Mon ML, Marrero Díaz de Villegas R, Campos E, Soria MA, Talia PM. Characterization of a novel GH10 alkali-thermostable xylanase from a termite microbiome. Bioresour Bioprocess. 2022. 10.1186/s40643-022-00572-w. 10.1186/s40643-022-00572-w PMC10992782 38647897

[108] Fredriksen L, Stokke R, Jensen MS, Westereng B, Jameson JK, Steen IH (2019). Discovery of a Thermostable GH10 Xylanase with Broad Substrate Specificity from the Arctic Mid-Ocean Ridge Vent System. Appl Environ Microbiol..

[109] Knapik K, Becerra M, Gonzalez-Siso MI (2019). Microbial diversity analysis and screening for novel xylanase enzymes from the sediment of the Lobios Hot Spring in Spain. Sci Rep.

[110] Rajabi M, Nourisanami F, Ghadikolaei KK, Changizian M, Noghabi KA, Zahiri HS (2022). Metagenomic psychrohalophilic xylanase from camel rumen investigated for bioethanol production from wheat bran using Bacillus subtilis AP. Sci Rep.

[111] Hu D, Zhao X (2022). Characterization of a new xylanase found in the rumen metagenome and its effects on the hydrolysis of wheat. J Agric Food Chem.

[112] Wang J, Liang J, Li Y, Tian L, Wei Y (2021). Characterization of efficient xylanases from industrial-scale pulp and paper wastewater treatment microbiota. AMB Express.

[113] Ariaeenejad S, Hosseini E, Maleki M, Kavousi K, Moosavi-Movahedi AA, Salekdeh GH (2019). Identification and characterization of a novel thermostable xylanase from camel rumen metagenome. Int J Biol Macromol.

[114] Ariaeenejad S, Maleki M, Hosseini E, Kavousi K, Moosavi-Movahedi AA, Salekdeh GH (2019). Mining of camel rumen metagenome to identify novel alkali-thermostable xylanase capable of enhancing the recalcitrant lignocellulosic biomass conversion. Bioresour Technol.

[115] Ariaeenejad S, Lanjanian H, Motamedi E, Kavousi K, Moosavi-Movahedi AA, Hosseini SG (2020). The stabilizing mechanism of immobilized metagenomic xylanases on bio-based hydrogels to improve utilization performance: computational and functional perspectives. Bioconjug Chem.

[116] Mousavi SH, Sadeghian Motahar SF, Salami M, Kavousi K, Sheykh Abdollahzadeh Mamaghani A, Ariaeenejad S, et al. Invitro bioprocessing of corn as poultry feed additive by the influence of carbohydrate hydrolyzing metagenome derived enzyme cocktail. Sci Rep. 2022;12(1):405. 10.1038/s41598-021-04103-z. 10.1038/s41598-021-04103-z PMC8749004 35013392

[117] Ariaeenejad S, Kavousi K, Zolfaghari B, Roy S, Koshiba T, Hosseini SG (2023). Efficient bioconversion of lignocellulosic waste by a novel computationally screened hyperthermostable enzyme from a specialized microbiota. Ecotoxicol Environ Saf.

[118] Pavarina GC, Lemos EGM, Lima NSM, Pizauro JM (2021). Characterization of a new bifunctional endo-1,4-beta-xylanase/esterase found in the rumen metagenome. Sci Rep.

[119] Ariaeenejad S, Kavousi K, Maleki M, Motamedi E, Moosavi-Movahedi AA, Hosseini SG (2021). Application of free and immobilized novel bifunctional biocatalyst in biotransformation of recalcitrant lignocellulosic biomass. Chemosphere.

[120] Ariaeenejad S, Motamedi E, Kavousi K, Ghasemitabesh R, Goudarzi R, Salekdeh GH (2022). Enhancing the ethanol production by exploiting a novel metagenomic-derived bifunctional xylanase/beta-glucosidase enzyme with improved beta-glucosidase activity by a nanocellulose carrier. Front Microbiol.

[121] Sanjaya RE, Putri KDA, Kurniati A, Rohman A, Puspaningsih NNT (2021). In silico characterization of the GH5-cellulase family from uncultured microorganisms: physicochemical and structural studies. J Genet Eng Biotechnol.

[122] Patel M, Patel HM, Dave S (2020). Determination of bioethanol production potential from lignocellulosic biomass using novel Cel-5m isolated from cow rumen metagenome. Int J Biol Macromol.

[123] Stepnov AA, Fredriksen L, Steen IH, Stokke R, Eijsink VGH (2019). Identification and characterization of a hyperthermophilic GH9 cellulase from the Arctic Mid-Ocean Ridge vent field. PLoS ONE.

[124] Hammami A, Fakhfakh N, Abdelhedi O, Nasri M, Bayoudh A (2018). Proteolytic and amylolytic enzymes from a newly isolated *Bacillus **mojavensis* SA: Characterization and applications as laundry detergent additive and in leather processing. Int J Biol Macromol.

[125] Nguyen KHV, Dao TK, Nguyen HD, Nguyen KH, Nguyen TQ, Nguyen TT, et al. Some characters of bacterial cellulases in goats’ rumen elucidated by metagenomic DNA analysis and the role of fibronectin 3 module for endoglucanase function. Anim Biosci. 2021;34(5):867–79. 10.5713/ajas.20.0115. 10.5713/ajas.20.0115 PMC8100471 32882773

[126] Guerrero EB, de Villegas RMD, Soria MA, Santangelo MP, Campos E, Talia PM (2020). Characterization of two GH5 endoglucanases from termite microbiome using synthetic metagenomics. Appl Microbiol Biotechnol.

[127] Maleki M, Shahraki MF, Kavousi K, Ariaeenejad S, Hosseini SG (2020). A novel thermostable cellulase cocktail enhances lignocellulosic bioconversion and biorefining in a broad range of pH. Int J Biol Macromol.

[128] Motamedi E, Sadeghian Motahar SF, Maleki M, Kavousi K, Ariaeenejad S, Moosavi-Movahedi AA (2021). Upgrading the enzymatic hydrolysis of lignocellulosic biomass by immobilization of metagenome-derived novel halotolerant cellulase on the carboxymethyl cellulose-based hydrogel. Cellulose.

[129] Ariaeenejad S, Sheykh Abdollahzadeh Mamaghani A, Maleki M, Kavousi K, Foroozandeh Shahraki M, Hosseini Salekdeh G. A novel high performance in-silico screened metagenome-derived alkali-thermostable endo-beta-1,4-glucanase for lignocellulosic biomass hydrolysis in the harsh conditions. BMC Biotechnol. 2020;20(1):56. doi: 10.1186/s12896-020-00647-6. 10.1186/s12896-020-00647-6 PMC7574624 33076889

[130] Chai S, Zhang X, Jia Z, Xu X, Zhang Y, Wang S (2020). Identification and characterization of a novel bifunctional cellulase/hemicellulase from a soil metagenomic library. Appl Microbiol Biotechnol.

[131] Yan Z, Ding L, Zou D, Wang L, Tan Y, Guo S (2021). Identification and characterization of a novel carboxylesterase EstQ7 from a soil metagenomic library. Arch Microbiol.

[132] Zhang Y, Ding L, Yan Z, Zhou D, Jiang J, Qiu J (2023). Identification and characterization of a novel carboxylesterase belonging to family VIII with promiscuous acyltransferase activity toward cyanidin-3-O-glucoside from a soil metagenomic library. Appl Biochem Biotechnol.

[133] Lu M, Daniel R (2021). A novel carboxylesterase derived from a compost metagenome exhibiting high stability and activity towards high salinity. Genes (Basel)..

[134] Ariaeenejad S, Kavousi K, Mamaghani ASA, Motahar SFS, Nedaei H, Salekdeh GH (2021). In-silico discovery of bifunctional enzymes with enhanced lignocellulose hydrolysis from microbiota big data. Int J Biol Macromol.

[135] Kaushal G, Rai AK, Singh SP (2021). A novel beta-glucosidase from a hot-spring metagenome shows elevated thermal stability and tolerance to glucose and ethanol. Enzyme Microb Technol.

[136] Thornbury M, Sicheri J, Slaine P, Getz LJ, Finlayson-Trick E, Cook J (2019). Characterization of novel lignocellulose-degrading enzymes from the porcupine microbiome using synthetic metagenomics. PLoS ONE.

[137] Salami M, Sadeghian Motahar SF, Ariaeenejad S, Sheykh Abdollahzadeh Mamaghani A, Kavousi K, Moosavi-Movahedi AA, et al. The novel homologue of the human alpha-glucosidase inhibited by the non-germinated and germinated quinoa protein hydrolysates after in vitro gastrointestinal digestion. J Food Biochem. 2022;46(1):e14030. 10.1111/jfbc.14030. 10.1111/jfbc.14030 34914113

[138] Ariaeenejad S, Zolfaghari B, Sadeghian Motahar SF, Kavousi K, Maleki M, Roy S (2021). Highly efficient computationally derived novel metagenome alpha-amylase with robust stability under extreme denaturing conditions. Front Microbiol.

[139] Sadeghian Motahar SF, Ariaeenejad S, Salami M, Emam-Djomeh Z, Sheykh Abdollahzadeh Mamaghani A. Improving the quality of gluten-free bread by a novel acidic thermostable alpha-amylase from metagenomics data. Food Chem. 2021;352:129307. 10.1016/j.foodchem.2021.129307. 10.1016/j.foodchem.2021.129307 33691209

[140] Thakur M, Sharma N, Rai AK, Singh SP (2021). A novel cold-active type I pullulanase from a hot-spring metagenome for effective debranching and production of resistant starch. Bioresour Technol.

[141] Sadeghian Motahar SF, Salami M, Ariaeenejad S, Emam‐Djomeh Z, Sheykh Abdollahzadeh Mamaghani A, Kavousi K, et al. Synergistic Effect of metagenome‐derived starch‐degrading enzymes on quality of functional bread with antioxidant activity. Starch Stärke. 2021; doi: 10.1002/star.202100098.

[142] Itoh N, Hayashi Y, Honda S, Yamamoto Y, Tanaka D, Toda H (2021). Construction and characterization of a functional chimeric laccase from metagenomes suitable as a biocatalyst. AMB Express.

[143] Ariaeenejad S, Kavousi K, Afshar Jahanshahi D, Sheykh Abdollahzadeh Mamaghani A, Ghasemitabesh R, Moosavi-Movahedi AA, et al. Enzymatically triggered delignification through a novel stable laccase: a mixed in-silico /in-vitro exploration of a complex environmental microbiota. Int J Biol Macromol. 2022;211:328–41. 10.1016/j.ijbiomac.2022.05.039. 10.1016/j.ijbiomac.2022.05.039 35551951

[144] Motamedi E, Kavousi K, Sadeghian Motahar SF, Reza Ghaffari M, Sheykh Abdollahzadeh Mamaghani A, Hosseini Salekdeh G, et al. Efficient removal of various textile dyes from wastewater by novel thermo-halotolerant laccase. Bioresour Technol. 2021;337:125468. 10.1016/j.biortech.2021.125468. 10.1016/j.biortech.2021.125468 34320748

[145] Verma SK, Sharma PC (2021). Isolation and biochemical characterization of a novel serine protease identified from solid tannery waste metagenome. Biologia.

[146] Verma SK, Kaur S, Tevetia A, Chatterjee S, Sharma PC (2021). Structural characterization and functional annotation of microbial proteases mined from solid tannery waste metagenome. Biologia.

[147] Ariaeenejad S, Kavousi K, Mamaghani ASA, Ghasemitabesh R, Hosseini SG (2022). Simultaneous hydrolysis of various protein-rich industrial wastes by a naturally evolved protease from tannery wastewater microbiota. Sci Total Environ.

[148] Wu S, Nan F, Jiang J, Qiu J, Zhang Y, Qiao B (2019). Molecular cloning, expression and characterization of a novel feruloyl esterase from a soil metagenomic library with phthalate-degrading activity. Biotechnol Lett.

[149] Cavello IA, Hours RA, Cavalitto SF (2013). Enzymatic hydrolysis of gelatin layers of X-Ray films and release of silver particles using keratinolytic serine proteases from *Purpureocillium lilacinum* LPS # 876. J Microbiol Biotechnol.

[150] Sarkar J, Dutta A, Pal Chowdhury P, Chakraborty J, Dutta TK (2020). Characterization of a novel family VIII esterase EstM2 from soil metagenome capable of hydrolyzing estrogenic phthalates. Microb Cell Fact.

[151] Kaur R, Kumar R, Verma S, Kumar A, Rajesh C, Sharma PK (2020). Structural and functional insights about unique extremophilic bacterial lipolytic enzyme from metagenome source. Int J Biol Macromol.

[152] Castillo Villamizar GA, Nacke H, Griese L, Tabernero L, Funkner K, Daniel R (2019). Characteristics of the first protein tyrosine phosphatase with phytase activity from a soil metagenome. Genes (Basel)..

[153] Karunatillaka I, Jaroszewski L, Godzik A (2022). Novel putative polyethylene terephthalate (PET) plastic degrading enzymes from the environmental metagenome. Proteins.

[154] Goncalves TA, Sodre V, da Silva SN, Vilela N, Tomazetto G, Araujo JN (2022). Applying biochemical and structural characterization of hydroxycinnamate catabolic enzymes from soil metagenome for lignin valorization strategies. Appl Microbiol Biotechnol.

[155] Sun J, Yao C, Li Y, Wang W, Hao J, Yu Y (2022). A novel salt-tolerant GH42 beta-galactosidase with transglycosylation activity from deep-sea metagenome. World J Microbiol Biotechnol.

[156] Creekmore BC, Gray JH, Walton WG, Biernat KA, Little MS, Xu Y (2019). Mouse gut microbiome-encoded beta-glucuronidases identified using metagenome analysis guided by protein structure. mSystems..

[157] Wierzbicka-Wos A, Henneberger R, Batista-Garcia RA, Martinez-Avila L, Jackson SA, Kennedy J (2019). Biochemical characterization of a novel monospecific endo-beta-1,4-glucanase belonging to GH family 5 from a rhizosphere metagenomic library. Front Microbiol.

[158] Yasir M, Khan H, Azam SS, Telke A, Kim SW, Chung YR (2013). Cloning and functional characterization of endo-beta-1,4-glucanase gene from metagenomic library of vermicompost. J Microbiol.

[159] Holck J, Djajadi DT, Brask J, Pilgaard B, Krogh K, Meyer AS (2019). Novel xylanolytic triple domain enzyme targeted at feruloylated arabinoxylan degradation. Enzyme Microb Technol.

